# Modulation of the Endocannabinoid System: Vulnerability Factor and New Treatment Target for Stimulant Addiction

**DOI:** 10.3389/fpsyt.2013.00109

**Published:** 2013-09-23

**Authors:** Stéphanie Olière, Antoine Jolette-Riopel, Stéphane Potvin, Didier Jutras-Aswad

**Affiliations:** ^1^Addiction Psychiatry Research Unit, Research Center, Centre Hospitalier de l’Université de Montréal (CRCHUM), Montreal, QC, Canada; ^2^Department of Psychiatry, University of Montreal, Montreal, QC, Canada; ^3^Research Center, Institut Universitaire en Santé Mentale de Montréal, Montreal, QC, Canada

**Keywords:** addiction, stimulants, psychostimulants, cocaine, cannabis, cannabinoids or endocannabinoids

## Abstract

Cannabis is one of the most widely used illicit substance among users of stimulants such as cocaine and amphetamines. Interestingly, increasing recent evidence points toward the involvement of the endocannabinoid system (ECBS) in the neurobiological processes related to stimulant addiction. This article presents an up-to-date review with deep insights into the pivotal role of the ECBS in the neurobiology of stimulant addiction and the effects of its modulation on addictive behaviors. This article aims to: (1) review the role of cannabis use and ECBS modulation in the neurobiological substrates of psychostimulant addiction and (2) evaluate the potential of cannabinoid-based pharmacological strategies to treat stimulant addiction. A growing number of studies support a critical role of the ECBS and its modulation by synthetic or natural cannabinoids in various neurobiological and behavioral aspects of stimulants addiction. Thus, cannabinoids modulate brain reward systems closely involved in stimulants addiction, and provide further evidence that the cannabinoid system could be explored as a potential drug discovery target for treating addiction across different classes of stimulants.

## Introduction

Addiction to psychostimulants such as cocaine, amphetamine, and its derivatives [i.e., methamphetamine, *N*-methyl-3,4-methylenedioxymethamphetamine (MDMA)] is a significant global public health problem which affects many aspects of social and economic life. Worldwide, between 16 and 51 million people are users of these types of substances (1). Amphetamines have been identified as the second world’s most widely used illicit drug after cannabis, with an annual prevalence ranging from 0.3 to 1.2% in the adult population. Methamphetamine consumption has increased dramatically the last years, especially in the western and mid-western parts of the United States, although there also appears to be an eastward trend in use (2). Over 15 million people worldwide are cocaine users and 5.9 million of them live in North America (3, 4). Although the prevalence of cocaine consumption has declined in the past decade, cocaine use increased in 2011; dependence to this drug remains a significant issue in North America, Western and Central Europe, particularly in metropolitan areas where crime and violence have increased (4). Given its association with high rates of mental and physical problems as well as premature mortality, cocaine abuse is still an unresolved medical and socio-economic concern which carries a heavy burden for abusers and their families alike (3, 5–7).

In recent decades, development of new treatments for psychostimulant addiction has been a major focus of multidisciplinary research efforts and have included molecular approaches, pre-clinical behavioral studies, and clinical trials. However, in spite of these research endeavors, no specific pharmacological therapy has been found to be truly effective in alleviating psychostimulant cessation symptoms like craving and anxiety, or to prevent relapse (8–11). Neuropharmacological agents such as antidepressants, anticonvulsants, and antipsychotics have been tested as treatments for cocaine dependence but these medications have yielded negative clinical outcomes (12–14). Subsequent attempts at targeting other neurotransmitters such as the dopaminergic and γ-aminobutyric acid (GABA) systems (10, 15, 16), and developing a cocaine vaccine (17) to promote abstinence in cocaine-dependent individuals, have shown promising results but still require further investigation. Importantly, many clinical studies have focused on cocaine addiction rather than other psychostimulants such as amphetamines and methylphenidate. Whether the outcomes related to cocaine addiction can be applied to other psychostimulants remains unclear (18).

Given the need to better understand neurobiological mechanisms that underly psychostimulants addiction and to develop innovative treatment strategies, researchers have explored the involvement of specific neurotransmitter systems and brain structures in the motivational and addictive properties of this class of drugs. Increasing evidence indicates that the endocannabinoid system (ECBS) - a group of neuromodulatory lipids and receptors – plays a central role in various cognitive and physiological processes associated with addiction such as reward, stress responsiveness, and drug-related synaptic plasticity (19–21). The potential of ECBS modulation in treating stimulant addiction has recently been highlighted in human and animal studies investigating its effects on acquisition, maintenance, and relapse of drug-taking behavior. Moreover, endogenous and exogenous cannabinoids such as plant-derived cannabinoid ligands (i.e., Δ^9^-THC, cannabidiol, CBD) modulate specific neurotransmitter systems which are also pharmacological targets for cocaine. Interestingly, cannabis is widely used by psychostimulant-dependent individuals (22); while it is recognized that components of the ECBS are implicated in psychostimulants, more specifically in cocaine-seeking behaviors, very few studies have focused on an understanding of the neural and behavioral effects of cannabis on psychostimulants use in humans.

The current review will focus on the neurobiological basis of the addictive process from psychostimulant initiation to drug abuse and addiction. The involvement of critical neurotransmitters and neural circuits underlying the pathological modifications at each of these stages will be highlighted with a specific attention given to the ECBS. In turn, this overview will serve as foundations to look at the ECBS as a specific target of pharmacotherapeutic interventions to reduce the addictive effects of psychostimulants.

## Neurobiology of Psychostimulants

Occasional use of psychostimulants like cocaine, at low doses, provokes a so-called “rush” (i.e., euphoria) in humans, giving them a sensation of vigilance and increased energy. Higher doses of cocaine induces symptoms described as “cocaine high,” which include enhancement of a euphoric sensation, an increase in motor activity, amplification of sensory perception, talkativeness, and suppression of appetite and thirst (23). Unfortunately, these positive subjective effects (i.e., euphorigenic state) are often followed by repetitive and frequent cocaine abuse which develops into addiction. Drug addiction is a chronically relapsing disorder characterized by loss of control over drug-seeking and the compulsive desire (referred as craving) to use drugs in spite of negative consequences (24). Drug cravings increase with exposure to drug and drug-related-cues, and in the context of emotional stress or negative moods (25, 26). Because addiction is a highly complex disorder, numerous studies have attempted to determine the molecular and cellular factors implicated in the pleasurable effects induced by drug consumption, and their role in the development of addictive behaviors (27–31).

### Neurotransmitters involved in processes leading to psychostimulants addiction

Psychostimulants affect the central nervous system by modulating the mesocorticolimbic dopamine (DA) system which is involved in several physiological processes such as cognition, memory, and reward-driven learning (32). The mesocorticolimbic system, which has been found to play a role in drug reward and addiction, includes DA projections from cell bodies in the ventral tegmental area (VTA) to limbic structures such as the nucleus accumbens (NAc) (33), amygdala in the forebrain (34, 35), hippocampus (36), and to cortical areas such as the prefontal cortex (PFC), including the orbitofrontal cortex (OFC) and anterior cingulate (AC) (37). Psychostimulants exert their effects on the CNS via a number of mechanisms; cocaine, amphetamines, methamphetamines, and methylphenidate alter normal DA receptor functions by binding the dopamine transporter (DAT) and forming a complex that blocks the transporter’s function. The psychostimulant/DAT complex inhibits DA reuptake into the presynaptic nerve terminal, leading to an excess of DA in the synaptic cleft within the NAc – recognized as the center of the rewarding process (38–40). This phenomenon results in an increased and prolonged post-synaptic effect of dopaminergic signaling at DA receptors on the receiving neuron (30, 31). However, unlike cocaine, which interferes mainly with plasma membrane transporters, other psychostimulants modulate the CNS through a host of mechanisms. First, methamphetamines and amphetamines act as substrate-type releasers (41, 42) to enhance DA efflux. These substrate-type releasers have two modes of action: (i) they reverse the process of transporter-mediated exchange by interacting with specific transporter proteins which are subsequently brought into the cytoplasm of the nerve terminal; (ii) they also increase cytoplasmic levels of DA by interfering with vesicular storage (43, 44). Moreover, these drugs increase cytosolic DA levels by shutting-down the activity of the monoamine oxidase (MAO) – an important enzyme for the catabolism of monoaminergic neurotransmitters. Finally, psychostimulants also enhance the activity and expression of the tyrosine hydroxylase (TH), the DA-synthesizing enzyme [reviewed in Ref. (45)]. However, exactly how these high levels of DA in the NAc mediates drug reward remains partially understood.

Even though DA has been identified as one of the primary mechanisms involved in drug reinforcement initiation, studies reveal that mice lacking the gene expressing the DAT continue to self-administer cocaine (46, 47). Interestingly, several reports have suggested the indirect implication of other neurotransmitter systems [i.e., serotonin (5-HT), norepinephrine (NE), glutamate (GLU), GABA, opioid peptides, and endocannabinoids (48–50)] in the incentive sensitization and reinforcing effects of psychostimulants (29, 51). Indeed, psychostimulants also reduce 5-HT and norepinephrine NE reuptake, which in turn leads to an increase in extracellular monoamines concentrations and contributes to the rewarding subjective feelings mediated by these drugs (42, 52–54). Surprisingly, knock-down of NET, or SERT, or NET/SERT genes does not abolish but rather potentiates the rewarding or aversive effects of cocaine (55, 56). Recently, further lines of evidence have suggested that NE plays a role in the reinstatement of drug seeking, although it does not influence the maintenance phase of cocaine self-administration (SA) (57–59). Blockage of NE cognate receptors – α1-adrenergic receptors (α1ARs) and β-adrenergic receptors (βARs) – in a mice model of addiction diminishes cocaine-primed and foot shock-induced reinstatement respectively, whereas inhibition of both receptors reduces cue-induced reinstatement (60, 61).

Long-term use of psychostimulants leads to homeostatic dysregulation of normal (i.e., without cocaine) dopaminergic signaling. This hypo-dopaminergic state contributes to the appearance of some withdrawal symptoms (i.e., depressive mood disorders), often observed in abstinent psychostimulant addicts, and, also the maintenance of drug-use behaviors. Similarly, withdrawal symptoms from chronic cocaine use have been also associated with cocaine-induced alterations in 5-HT neurotransmission. Interestingly, rodent studies show that enhancement of serotonergic transmission in the NAc through administration of exogenous 5-HT served to offset the DA deficit caused by cocaine withdrawal (62); indeed, accumulating studies suggest that increasing brain 5-HT activity could reduce the behavioral-stimulant and reinforcing properties of psychostimulants (reviewed in Ref. (44)]. Thus, modulation of 5-HT and DA levels might sensitize an important brain reward circuit to the reinforcing effects of psychostimulants contributing to the intractable nature of addiction and relapse.

The glutamatergic system is another important neuronal substrate of behaviors induced by drugs of abuse (63, 64). Indeed, GLU is an excitatory neurotransmitter essential to numerous processes including neuroplasticity, linked to long-term potentiation (LTP), long-term depression (LTD), extinction, and reward-related learning (65–68). Like DA, GLU levels in the NAc core decrease during the early phase of cocaine abstinence (69, 70), whereas both stress and drug-induced reinstatement of cocaine-seeking are associated with an increase of extracellular GLU levels in the NAc in rodents (63, 64, 70–73). Thus, the data suggests that both a decrease in basal GLU transmission and an enhanced GLU response may constitute a neurobiological substrate of cocaine-, cocaine-associated cue-triggered relapse.

While discovery of numerous neurotransmitter systems have yielded significant advances in defining psychostimulants’ effects on the brain neurochemistry, the precise mechanisms underlying their role in addictive behaviors are not as straightforward. While DA and GLU appear to be critical in the development and persistence of stimulant addictive behaviors, a growing body of evidence points toward the impact of other neurotransmission systems, including the ECBS, in various physiological and behavioral processes associated with psychostimulant addiction, through both DA/GLU related and unrelated mechanisms. An overview of this evidence will be presented in Section “The Endocannabinoid System,” with a particular focus on the potential exogenous and endogenous cannabinoids influence on psychostimulant reinforcement, drug-related synaptic plasticity, and drug-seeking behavior.

### Neural regions involved in addiction process to psychostimulants

A central challenge in addiction research is understanding the neurobiological substrates involved in drug-taking behavior. Over the last two decades, neuroimaging has provided substantial insight into that question by: (i) allowing researchers to investigate the roles of different neural regions in drug-induced euphoria and subsequent craving; (ii) enabling the gathering of tremendous information regarding the neurochemical and physiological adaptations of the brain during the addiction process.

Brain imaging studies of subjects addicted to psychostimulants indicate that the NAc – known to play a fundamental role in goal-directed behaviors (74) – is organized into two functionally distinct sub-compartments termed the shell and core (33). The shell and the VTA are critical in inducing motivational salience and responding to novel rewarding stimuli (75). The core mediates the expression of learned behaviors, and receives glutamatergic afferents from the PFC (33, 75). DA release into the core occurs in response to cues predicting a motivating event (76, 77). The NAc receives information regarding motivationally relevant events from the VTA, amygdala, hippocampus, and PFC, and responds by providing output to brain circuits which modulate the expression of the behavioral response (e.g., to seek the drug or not) (78). Chronic exposure to psychostimulants leads to the dysregulation of the mesolimbic circuitry, which in turn enhances the motivation to take drugs and decreases the ability to regulate the behavioral response to drug cues (33, 74).

The numerous neuroimaging methods used to study the chronic effects of psychostimulants on the brains of drug-addicted individuals have consistently found abnormalities in both cortical and subcortical neural areas (37, 79). More specifically, chronic exposure to psychostimulants causes functional alterations within frontal brain areas, including the dorsolateral prefrontal cortex (DLPFC), the OFC, and the anterior cingulate cortex (ACC) involved in goal identification; selection (80); decision making; impulsivity; behavioral inhibition (81), and assessment of consequences (82), respectively. It has been proposed that abnormalities within these three PFC-striatothalamic circuits play a central role in emotional response to drug cues, craving, compulsive drug-seeking, and relapse (26, 35, 83–85). Moreover, structural magnetic resonance imaging (structural MRI) studies associate chronic use of psychostimulants with alterations in white-matter integrity and gray-matter volume, which are strongly correlated with lower abstinence-based outcomes (86) and drug-induced compulsivity, decision making, and attention impairments in cocaine-dependent subjects, respectively (85). Furthermore, exposure to emotional distress and aversive stimuli also activates the cortico-limbic circuits, including prefrontal, AC, middle frontal, and orbitofrontal regions, limbic and paralimbic structures such as the amygdala, hippocampus, parahippocampal gyrus, fusiform gyrus, and other midbrain areas, but not the ventral striatum (87, 88). Overall, the data shows that chronic use of psychostimulants modulates a set of neural regions implicated in stress, emotions, impulsivity, and reward processing control which precipitate relapse in drug-abstinent individuals.

## The Endocannabinoid System

Though the significant role played by various neurotransmitters, genetic factors and specific brain structures in reinforcing the properties of psychostimulants has been established, the common mechanisms underlying the development of addictive behaviors have yet to be fully elucidated. A growing body of evidence points to the involvement of the ECBS in the acquisition and maintenance of drug-taking behaviors and in various physiological, as well as behavioral processes associated with addiction. Interestingly, a characteristic of psychostimulants abuse is the concurrent consumption of other substances including delta-9-tetrahydrocannabinol (Δ^9^-THC) – the main cannabinoid found in cannabis [reviewed in Ref. (89)]. This poly-substance pattern of use has prompted researchers to investigate the potential interaction with, and effect of, these drug on neuropsycho-biological processes related to addiction. For example, some studies reveal that cannabis consumption enhances the incentive to use cocaine in individuals dependent on both, while others suggested that cannabis reduced withdrawal symptoms in abstinent cocaine-addicted subjects (22, 90, 91). Although it is recognized that components of the ECBS are involved in cocaine-seeking behaviors, very few studies have focused on understanding the neural and behavioral effects of endogenous and exogenous cannabinoids on psychostimulant use. In this section, we will first provide an overview of the ECBS, and then focus on recent findings pointing toward a role of the ECBS in the circuitry underlying psychostimulant addiction.

### Overview

The ECBS consists of a family of lipid signaling molecules referred to as endocannabinoids, their cognate receptors and specific metabolic enzymes which are responsible for degradation of the endocannabinoids – anandamide (AEA) and 2-arachidonoylglycerol (2-AG). The neurobiological properties of endocannabinoids are complex, but it is now well established that they modulate a wide diversity of physiological processes including pain and inflammation, immune responses, food intake, synaptic transmission, cognition, reward, and motor activity (92). Endocannabinoids also influence mechanisms involved in addiction and relapse.

### Receptors

There are currently two well described subtypes of cannabinoid receptors, termed CB1 and CB2, which differ in their signaling mechanisms and tissue distribution. Even though CB1 receptors are considered the most abundant and widely distributed G-protein-coupled receptors found in the CNS, they are also present in peripheral organs and tissues (i.e., endocrine glands, leukocytes, spleen, heart, and gastrointestinal tracts, etc.) (93–97). CB1 receptors are localized in TH-expressing neurons, probably dopaminergic neurons of the NAc, VTA, striatum, and pyriform cortex, suggesting that the ECBS may directly influence dopaminergic reward mechanisms. In addition, CB1 receptors are expressed in other neural regions related to reward, motivation and memory processing (i.e., basolateral amygdala, hippocampus, and cerebral cortex), movement (i.e., basal ganglia, cerebellum), pain modulation (i.e., certain parts of the spinal cord, periaqueductal gray). Endocannabinoids induce LTD of the inhibitory synapses in the hippocampus, contributing to the synaptic plasticity involved in the learning processes related to addictive behaviors. CB1 receptors are confined at the terminals of central and peripheral nerves, where they inhibit the release of excitatory and inhibitory neurotransmitters (release on command, retrograde signaling) (98–100). Thus, the activation of CB1 receptors protects the nervous system from over-activation or over-inhibition by neurotransmitters and thereby promotes the latter’s prominent role in anxiety, depression, cognition, addiction, motor function, feeding behavior, and pain (101). CB2 receptors are mainly found in immune cells (i.e., spleen, tonsils, and thymus gland) (102–104), although recent experimental data indicate CB2 receptors expression in the cerebellum, brainstem, and cortex (105–107) as well as activated microglial within the CNS (108–110). Simulation of CB2 receptors on microglia modulates the neuro-inflammatory response by regulating cytokines release in the brain (111–113).

Increasing evidence points toward the existence of additional cannabinoid receptors subtypes in the CNS. Indeed, recent pre-clinical studies suggest the persistence of cannabinoid-like properties after cannabinoid agonists have been administered to mice lacking CB1 and CB2 receptors (CB1^−/−^ and CB2^−/−^) in neuronal subpopulations. This indicates that these agonists recognize non-CB1/CB2 cannabinoid receptors (114–117). Among these receptors, the orphan G-protein-coupled receptors modulate the ECBS. GPR55 specifically is found in the striatum and to a lesser extent in the hippocampus, the thalamus and the cerebellum (118). GPR55 is phylogenetically different from CB1 and CB2 receptors, in that it is activated by the CB1 antagonists – rimonabant and AM251 – but blocked by the cannabinoid agonist CP55, 940 (119–121). Thus, GPR55 is considered as a non-CB receptor with a binding site for cannabinoid ligands [reviewed in Ref. (122)]. Though a recent study from Rusakov’s group suggests that GPR55 enhances neurotransmitters release at central synapses (123), further studies are required to confirm its neurophysiological function.

The actions of endocannabinoids are not only restricted to the CB1, CB2, and GPR receptors. Transient receptor potential (TRP) receptors have also been identified as sites of endocannabinoid interaction. Exogenous and endogenous cannabinoids interact with at least five TRP receptors (124); AEA binds to the transient receptor vanilloid potential 1 (TRVP1) with low affinity. TRVP1 is found on sensory neurons, where they are partly coexpressed with CB1 receptor, but also in several central nuclei including the hypothalamus and basal ganglia, the hippocampus and cerebellum (125). The efficacy and potency of AEA at TRVP1 is increased when the AEA degrading enzyme FAAH (fatty acid amide hydrolase) is suppressed (126–128). Surprisingly, pharmacological or genetic inhibition of FAAH enhances AEA, but decreases 2-AG levels via TRVP1 receptors (129). Interestingly, both endocannabinoids AEA and 2-AG decrease the excitatory GLU and the inhibitory GABAergic inputs to striatal neurons (130, 131). Therefore, it is likely that the potential of AEA to reduce 2-AG levels by activating TRVP1 receptors might represent a mechanism to integrate excitatory and inhibitory inputs in the basal ganglia.

### Endocannabinoids and their metabolizing enzymes

In the CNS, endocannabinoids mediate forms of short-term synaptic plasticity known as depolarization-induced suppression of inhibition (DSI) (132, 133) and depolarization-induced suppression of excitation (DSE) (134). Thus, endocannabinoids are considered as retrograde messengers that neuromodulate diverse physiological processes. AEA and 2-AG are the two most characterized endocannabinoids (135, 136), although other studies have identified of additional endocannabinoids such as 2-arachidonylglyceryl ether (noladin ether) (137), *N*-arachidonoyl-dopamine (NADA) (128), and *O*-arachidonoyl-ethanolamine (virodhamine) (138). However, the physiological functions of these endocannabinoids are still being investigated. While 2-AG acts as a full agonist at CB1 and CB2 receptors, AEA behaves as a partial agonist at both receptors subtypes and can also interact with GPR55 and TRVP1 receptors.

Unlike other neurotransmitters, AEA and 2-AG are not synthesized and stored in the nerve cells. Rather, they are produced on an “as needed” basis by their membrane lipid precursors in a Ca^2+^ dependent fashion (133, 139). Although additional studies are needed to ascertain the exact role of the *N*-acylphosphatidylethanolamine phospho-lipase D in the ECBS, it has been proposed that this enzyme might play a significant role in the synthesis of AEA (140). The enzyme responsible for 2-AG synthesis is the diacylglycerol lipase alpha (141). Upon depolarization of post-synaptic neurons, the endocannabinoids released into the synaptic cleft bind to and activate the presynaptic CB1 receptors, which in turn suppress the release of both excitatory and inhibitory different neurotransmitters [see for review (142)]. Then, AEA and 2-AG are rapidly deactivated by cellular reuptake into both neurons and glial cells and metabolized by specific enzymes (143). AEA can be metabolized by either the FAAH (144), or monoacylglycerol lipase (MAGL), which degrades specifically 2-AG (145). In addition to MAGL, recent studies have suggested that the enzymes ABHD6 and ABHD12 could also be involved in 2-AG metabolism (146, 147). FAAH is over-expressed in the CNS and FAAH-positive neurons are localized in proximity to CB1 receptor-containing terminals, underlining the role for this enzyme in endocannabinoids inhibition (148). Thus, selective inhibition of FAAH (149) and MAGL (150) can prolong the effects of endocannabinoids. Pre-clinical studies have demonstrated that pharmacological inhibition of FAHH with URB597 (149) or PF-3845 compounds (151) induced-anxiolytic-like effects (152, 153) and anti-nociceptive properties in mice (152, 154). Inhibition of MAGL with JZL184 inhibitor causes analgesia, hypothermia, and hypomotility (155). However, chronic exposure to JZL184 impairs endocannabinoid-mediated synaptic plasticity in mouse hippocampus and cerebellum via 2-AG upregulation. It also induces tolerance to the analgesic effects, physical dependence, and persistent activation as well as desensitization of brain CB1 receptors (156). Surprisingly, MAGL knockout mice show enhanced learning behavior and have normal locomotor activity, suggesting the possible role of MAGL in cognitive function (157, 158).

### Exogenous cannabinoids: Δ^9^-THC vs. CBD

Cannabis is the world’s most commonly used illicit drug (159, 160). Between 119 and 224 million people are cannabis users worldwide (4). Cannabis contains over 85 different chemical substances unique to the plant and termed phytocannabinoids. Among them, Δ^9^-tetrahydrocannabinol (Δ^9^-THC) and CBD are the two main components of cannabis, which has been used for thousands of years for both recreational and medicinal purposes. Most studies regarding cannabis properties have focused on Δ^9^-THC, which is the main psychoactive constituent in cannabis extracts (161). Although Δ^9^-THC possesses a number of therapeutic effects (e.g., on pain, spasms, inflammation), its negative impact on the CNS has been highlighted in several clinical studies on subjects smoking cannabis, documenting impulsive behavior, cognitive impairment, consumption of addictive substances, and psychiatric disorders (e.g., schizophrenia, depression, and anxiety) (162– 165). For example, Δ^9^-THC has been shown to induce psychotic-like and anxiogenic effects when administered intravenously to healthy subjects (166, 167). Other experimental studies revealed that Δ^9^-THC injection in animal models causes hypolocomotion, catalepsy, antinociception, and hypothermia (168).

Pharmacological studies in animal models suggest that not all therapeutic effects related to cannabis administration can be ascribed to Δ^9^-THC [reviewed in Ref. (169)]. Indeed, CBD – the second most abundant cannabinoid found in cannabis – acts as an antidepressant and possesses anticonvulsant, antiemetic, anxiolytic, and sleep-promoting as well as neuroprotective properties in humans (160, 170–176). CBD mediates its neuropharmacological properties by acting as an inverse agonist on CB1 and CB2 receptors (177, 178); it also stimulates the TRVP1 and TRVP2 (179) which serve as so-called ionotropic cannabinoid receptors. In addition, CBD inhibits FAAH, the main catabolic enzyme that alters the hydrolysis of the endogenous cannabinoid neurotransmitter AEA (180) (see above section), and is also an antagonist at the putative GPR55 receptor. The clinical association of the modulation of the ECBS by CBD remains to be fully investigated; this effect could arguably be related to DA uptake inhibition (181). Interestingly, ECBS interacts closely with other neurobiological structures which are implicated in the neural adaptations observed during chronic use of drugs and vulnerability to addiction. For example, CBD plays a role in the modulation of extracellular levels of DA (182) as well as μ and δ opioid receptors (183); it increases adenosine signaling through inhibition of uptake (184). Moreover, μ opioid and CB1 receptors colocalized within neural regions are known to modulate reward, goal-directed behavior, and habit formation relevant to addiction including striatal output projection neurons of the NAc and dorsal striatum (185, 186). While further studies are required to better understand the impact of CBD on GLU neurotransmission, its protective effects on GLU toxicity (187) and its psychopharmacologic interaction with ketamine (188), a *N*-methyl-d-aspartic (NMDA) receptor antagonist, are well documented. CBD activates also the serotoninergic receptors 5-HT1A (5-hydroxytryptamine) (171, 176, 189–193), which in turn diminishes vulnerability to stress and has anxiolytic-like effects in animal models (170, 172, 189, 190, 192–195). Similar results were observed in humans, where CBD administration decreases autonomic arousal and subjective anxiety (196). Interestingly, these anxiolytic effects have been linked to the modulation of core regions involved in the “emotional brain,” including limbic system structures such as the AMG and the ACC (197, 198). CBD’s anxiolytic effects were further confirmed by a study indicating that the effective connectivity between ACC and AMG is attenuated during the emotional processing of fearful faces, while resting activity of the left parahippocampus gyrus is increased. (196, 199). Remarkably, these neural structures are activated during drug craving in cocaine addiction (197, 200). It also decreases compulsive behaviors in rodents, which is hypothesized to be related to CB1-related mechanisms (201, 202).

While CBD has neuroprotective properties (187, 203, 204) and Δ^9^-THC administration have been shown to cause neurotoxic effects (205), these opposing properties have been highlighted in brain imaging studies where Δ^9^-THC and CBD activate different brain regions during tasks engaging verbal memory (206, 207), response inhibition (208), and emotional processing (196, 209–211). When given at appropriate doses, CBD counteracts Δ^9^-THC properties. Thus, CBD can modulate the functional effects of Δ^9^-THC (177, 178). Pre-clinical studies demonstrate that CBD decreases Δ^9^-THC-induced conditioned place aversion and social interaction of on operant behavior model (212, 213). In addition, CBD diminishes Δ^9^-THC-induced anxiety and psychotic-like symptoms in humans (214, 215). Together, this data clearly suggests that CBD limits Δ^9^-THC adverse effects. Thus, administered together, CBD might increase Δ^9^-THC clinical efficacy (216, 217). It has been established that unlike, Δ^9^-THC, CBD possesses therapeutic properties that could reduce withdrawal symptoms often present in individuals with addictive disorders (e.g., anxiety, psychotic, mood symptoms, insomnia, and pain). For example, a recent pre-clinical study from Hurd’s group aimed at assessing the effects of cannabinoids on opioid-seeking behaviors in rats indicates that while Δ^9^-THC potentiates heroin SA, CBD inhibits cue-induced heroin-seeking behaviors for up to 2 weeks following the last administration (218). In addition, CBD is well tolerated and has no gross effects on motor function (such as locomotor activity). CBD is also protects against damages caused by various substances; it reverses binge ethanol-induced neurotoxicity (219) and mitigates the cardiac effects of Δ^9^-THC (220, 221). Together this data illustrates the different, and sometimes opposite, neurobiological properties of the two main constituents of cannabis – CBD and Δ^9^-THC – that are linked to neural circuits which might play significant roles in addiction disorders. However, while numerous studies have highlighted the participation of the ECBS in the rewarding and addictive properties of drugs of abuse such as opioids, nicotine, and alcohol over the last decades, relatively few studies have focus on the impact of this system on addiction to psychostimulants.

## Interaction of the eCBS with Biological and Behavioral Correlates of Psychostimulants Addiction

### Human studies

Human studies aimed at understanding the interaction of the ECBS with biological and behavioral correlates of addiction to psychostimulants have mostly focused on ECBS-related risk factors leading to drug dependence. Interestingly, cannabis use is strongly associated with the abuse and/or dependence of several class of drugs including psychostimulants such as cocaine (222). Moreover, exogenous cannabinoids have been shown to modulate the acute rewarding effects of cocaine. These lines of evidence may suggest an association between ECBS and liability to psychostimulant by pointing toward a possible involvement of the ECBS in the motivational effects mediated by psychostimulants (223) [reviewed in Ref. (224)]. Based on these observations, scientific efforts have been devoted to investigate the influence of various genetic (e.g., ECBS-related genes) and environmental characteristics (e.g., previous or current exposure to cannabinoid agonists) in individual progression from occasional use to psychostimulant addiction.

#### “The gateway theory” and addiction to psychostimulants

Association of prior or concomitant cannabis consumption with other illicit drugs including psychostimulants such as methamphetamine and cocaine, forms the basis of a well-known hypothesis – “ the gateway theory,” which suggests a causal role for cannabis in the development of subsequent drug use and addiction (225). While data indicate that smoking cannabis is positively associated with cocaine consumption, it would be inappropriate to assume that cannabis *per se* leads to cocaine use. A study from Lynskey et al. in human twins reveals that early cannabis use in life increases the odds of subsequent cocaine use, supporting the causative model of the “gateway theory.” However, results of this study have been refuted by Kandel et al. (226) which argues that several additional genetic, social, and environmental factors, such as life experiences, might link cannabis use with subsequent cocaine consumption (227, 228). Actual neurobiological causal mechanisms underlying this “gateway theory” remain mostly unidentified. Interestingly, Tomasiewicz and colleagues show that Δ^9^-THC exposure induces epigenetic dysregulation of the endogenous opioid proenkephalin in adolescents; these findings indicate that cannabis exposure, in and of itself, can be considered as a risk factor that acts “above the genome” and can “write” on the existing epigenetic background of adolescent neurodevelopment. Thus, in adolescents, Δ^9^-THC exposure-mediated epigenetic effects may act in concert with other environmental or social factors to augment future behavioral responses to drugs of abuse via stable and long-term regulation of genes at the transcriptional level. However, while these data establish a direct link between Δ^9^-THC-induced changes in proenkephalin expression and susceptibility to opiate drugs, no studies have confirmed that this mechanism can be applied to psychostimulants (229).

#### Genetic determinants of the ECBS and psychostimulant addiction

It is worth mentioning that not every subject who experiences the pleasurable effects of psychostimulants will become a chronic user. Indeed it is more likely that additional factors such as: (1) genetic variabilities (e.g., polymorphisms in the catechol *O*-methyltransferase gene (Val158met) and in the serotonin transporter gene (5-HTTLPR) (230, 231); (2) monoamine receptors deficiency – either genetically or as a result of their drug excesses – also contribute to the psychostimulants addiction process (232–235) (see Table 1). In the ECBS, different genetic variants of the CB1 receptors – *CNR1* – and *FAAH* genes have been associated with increased susceptibility to drug addiction. Indeed, genetic analyses demonstrate that the *CNR1* gene exhibits elevated numbers of (AAT)n triplet repetition in a sample of 192 non-Hispanic Caucasian subjects. Interestingly, this *CNR1* polymorphism increases the risk of intravenous drug use in this population, with strongest correlation observed in cocaine, amphetamine, and marijuana dependence (236). Similarly, a study from Ballon and colleagues shows that detection of this *CNR1* polymorphism in a sample of 142 African-Caribbean individuals predisposed them to cocaine addiction (237). Unfortunately, while single sequence repetitions can alter transcriptional rates and thereby induced gene overexpression or silencing (238), the functional nature of the microsatellite polymorphism triplet repetition (AAT)n in modulating *CNR1* gene expression remains blurred (239). It has been hypothesized that the presence of long alleles with high numbers of AAT triplets alter *CNR1* transcriptional gene expression, ultimately leading to low levels of CNR1 protein synthesis (240). A recent meta-analysis of 11 studies aimed at investigating the contribution of three *CNR1* polymorphisms (rs1049353, rs806379, and the AAT triplet repetitions) to drug dependence vulnerability confirmed the presence of (AAT)n repeats, but only in the Caucasian population [reviewed in Ref. (239)]. Unfortunately, the effect of the three *CNR1* polymorphisms appeared to be insignificant and showed high heterogeneity. Important caveats have to be considered when looking at these studies. First, the ethnicity of the different subjects may prove important, as some studies included several ethnic groups in their samples, and in some cases, these groups were not even mentioned (241, 242). Some reports also examined *CNR1* gene polymorphisms in connection with a different phenotype or stage of drug addiction such as craving, drug consumption, dependence, or drug withdrawal (243). Furthermore, a detailed description of the repercussions of *CNR1* polymorphisms on CB1 function from a neurobiological standpoint is lacking from the reviewed studies.

**Table 1:** **ECBS and factors contributing to vulnerability to psychostimulants in humans**.

Aspect	Conclusion	Reference
Genetic risks factors	CNR1 (AAT)n repeat polymorphism associated with IV drug use, including amphetamine and cocaine, in a non-Hispanic Caucasian population and with cocaine dependence in an African-Caribbean population	Comings et al. (236), Ballon et al. (237)
	CNR1 gene single nucleotide polymorphisms associated with cocaine addiction in an African-American population	Clarke et al. (244)
	FAAH gene mis-sense mutation associated with drug dependence	Sipe et al. (245), Flanagan et al. (246)
Cannabis effect in addiction to psychostimulants	Self-reported of cannabis smoking by crack-cocaine abusers alleviates withdrawal symptoms and drug-craving	Labigalini et al. (90)
	Post-discharge use of cannabis by American cocaine addicts increases risk of relapse	Aharonovich et al. (91)
	Cannabis use correlates with syringe sharing in injection drug users	Jutras-Aswad et al. (22)
	Recent cannabis use decreases activation of frontal cortices area during emotional stress stimulation in cocaine-dependent individuals	Li et al. (247)

Polymorphisms in the gene coding for the endocannabinoid-inactivating enzyme *FAAH* may constitute another risk factor for problematic drug use, as described by initial reports identifying C385A, a mis-sense single nucleotide polymorphism (SNP) causing reduced FAAH enzymatic activity (245, 246). Indeed, a study from Sipe et al. reveals significant association between C385A SNP and street drug abuse in a sample of 1737 Caucasian subjects with addictive disorders. Neuroimaging studies combined with genetic analysis reveal that low FAAH activity enhances AEA protein expression levels which, in turn, modulate brain regions implicated in drug addiction and reward circuitry such as the OFC, AC gyrus, and NAc (242). Additional neuroimaging studies show that C385A carriers exhibit increased ventral striatal reactivity – a correlate for heightened impulsivity and reward sensitivity. C385A carriers display low threat-related amygdala reactivity – a pattern observed in individuals with high familial risk of alcoholism. Moreover, C385A polymorphism-reduced FAAH functional activity increases risk-taking behavior associated with addiction through abnormal impulsivity and threat perception [reviewed in Ref. (224, 243)]. Contribution of SNPs that modulate FAAH functions to stimulant addiction remain to be explored as the aforementioned data were not obtained in individuals specifically addicted to stimulants.

#### Effect of exogenous cannabinoids on psychostimulant reward

As mentioned previously (see The Endocannabinoid System), an intriguing characteristic of psychostimulants abuse is the concurrent consumption of cannabis. Parallel to studies on the long-term effects of cannabis exposure on subsequent psychostimulant use, researchers also examined the acute rewarding effects of cannabis use on concurrent psychostimulant addiction (91, 222). However, studies aimed at investigating such interactions are sparse. Conflicting results from Foltin et al. and Lukas et al. provide evidence that cannabinoids modulate cocaine-mediated euphoric actions. First, data from Foltin and colleagues show that human volunteers who smoked cannabis prior to intravenous cocaine experience a prolongation of the “high” sensation (248). Second, a study from Lukas and colleagues reveals that smoking Δ^9^-THC, 30 min prior to intranasal cocaine decreases the latency to onset of cocaine-induced euphoria significantly, from 1.87 to 0.53 min, as well as the duration of cocaine-induced dysphoria, from 2.1 to 0.5 min (249). Interestingly, when both drugs are administered concomitantly, no changes are observed in cocaine- and Δ^9^-THC-induced positive subjective properties. Furthermore, Δ^9^-THC increases the peak plasma levels and bioavailability of cocaine considerably. This increase might be the result of Δ^9^-THC-induced vasodilation of the nasal mucosa which, in turn, reduces cocaine-induced vasoconstriction, thereby increasing cocaine’s absorption. In addition, the discrepancies between these two studies might be also due to pharmacodynamic mechanisms including differences in cocaine absorption or in the ratio of CBD/Δ^9^-THC levels found in the type of cannabis used for each study.

Using fMRI technology combined with script-guided imagery paradigm in which subjects imagined being in a real-life stressful situation, Rajita Sinha’s group found that cannabis abuse contributed to stress-induced blood-oxygen-level-dependent (BOLD) contrast in a group of cocaine-dependent individuals. More specifically, cannabis consumption decreases emotional stress cue-induced frontal and cingulate activation in cocaine-dependent individuals (247). These findings suggest an abnormal cognitive control mechanism during affective processing in association with heavy cannabis use. An important caveat to consider in the latter study is that cocaine-dependent individuals were abstinent for several weeks prior to the neuroimagery session and were not current users of cannabis. Thus the study did not allow to examine the acute effect of cannabis on neural and behavioral responses. However, the fact that this cannabis-induced alteration in stress-response can be translated to cocaine craving and relapse vulnerability has definitely piqued further interest, and initial data on this matter already exists. Indeed, the effects of cannabis consumption on abstinence and relapse to cocaine use have been provided in a study from Labigalini and colleagues, in which 25 cocaine-crack dependent individuals reported to smoke cannabis in order to get relief from abstinence mediated-cocaine-withdrawal symptoms. From this sample, 68% of addicts achieved crack-cocaine cessation while using cannabis during the 9 months duration of the study (90). However, the self-reported nature of this study and its limited duration suggest cautiousness in interpreting its outcome. In a more recent study, Aharonovich et al. drew opposite conclusions on the consequences of smoking cannabis on cocaine relapse. In this study, researchers investigated whether cannabis use after the discharge of 144 drug-addicts from inpatient treatment program could help them to maintain abstinence and thereby preventing relapse to cocaine use. Results from this study suggest that smoking cannabis reduced the achievement of sustained remission and increased relapse to cocaine use (91) (see Table 1). Surprisingly, a study from Jutras-Aswad et al. supports the assumption that irregular cannabis use increases risky behaviors (syringe sharing) of cocaine and opioid users, as opposed to regular cannabis use, suggesting a complex dose-effect relationship between cannabis and addictive behaviors (22). The possibility that cannabis use by recently abstinent cocaine-dependent individuals influences relapse to drug and other related behaviors remains poorly documented.

### Animal studies

Over the last decades, development of animal models have allowed a better understanding of psychostimulant effects and addiction-related behaviors. These studies would not be available through clinical studies for ethical and practical reasons. Notably, invasive measures such as catheter installation for drug administration and surgical brain procedures for assessment of drug-induced neurobiological changes, as well as strictly controlled conditioning protocols involving restrictive environments, have extended the knowledge of psychostimulants effect on neurotransmission. These methods have also allowed observations of specific behavioral aspects of psychostimulant addiction. Thus, studies on animal models of psychostimulants abuse have provided tremendous insights on the role of ECBS in various aspects of psychostimulant addiction, spanning from drug reward, acquisition, and relapse.

#### Influence of ECBS on psychostimulants-induced behavioral and reinforcing effects

As mentioned above (see Neurobiology of Psychostimulants), substantial evidence indicates that behavioral and addictive properties of psychostimulants come from the interactions of psychostimulants with brain monoamines. Specifically, increase of extracellular levels of DA through promotion of DA release by amphetamine and MDMA, as well as inhibition of DA reuptake by cocaine, represent the primary mechanisms involved in rewarding effects mediated by psychostimulants (224). In animal models of intracranial self-stimulation (ICSS), the rewarding properties of drugs of abuse typically translate into lowering of the so-called reward threshold established after operant training [see Ref. (250) for description]. Initial experiments showed no effect of CB1 antagonist SR141716A on cocaine’s ability to lower ICSS threshold in rats (251), although careful data analysis suggests a tendency toward attenuation. However, different results were obtained when using a more potent antagonist – AM251. This antagonist proves CB1 blockade’s effectiveness in inhibiting cocaine’s action on brain stimulation reward (250). Paradoxically, the non-selective cannabinoid agonist – WIN55, 212-2 – and the endocannabinoid transmission enhancer – AM404 – are also able to abolish cocaine’s reinforcing effects as assessed by ICSS (252). Whether these apparently contradictory findings may indicate an inverse U-shape effect of CB1 stimulation function on rewarding properties of stimulants is not entirely clear. However, these results clearly indicate that cannabinoids might interfere with brain systems responsible for psychostimulants rewarding effects, and the mechanism underlying this phenomenon should be further explored.

Li and colleagues recently found significant reductions in DA levels in striatum of mice lacking the *CNR1* gene, when compared to their wildtype counterparts following acute cocaine administration and during the basal state (253). This observation shows consistency with above-cited ICSS studies and with a previous report on the inhibition of cocaine-induced DA release in rats by CB1 antagonist SR141716A (254). In contrast, initial findings suggested that neither basal levels nor cocaine-induced increases in extracellular NAc DA of CB1 knockout mice differed from that of normal mice (255), and that CB1 inactivation by antagonists AM251 and SR141716A failed to alter the increase in extracellular NAc DA responsible for cocaine-mediated rewarding effects in rats (256, 257). Differences in experimental methods used to measure DA levels (voltammetry vs. *in vivo* microdialysis) and in the genetic background of the knockout animals (C57BL/6J vs. CD1) could account for such discrepancies. Notably, compensatory neurobiological changes due to lack of CB1 receptors could explain the subnormal basal DA levels observed in Li et al. study. This subnormal basal DA levels could also have contributed to apparent attenuation of DA levels enhancement produced by cocaine. Overall, the extent to which ECBS interaction with psychostimulants-mediated reward effects depends on DA transmission seems limited, especially when CB1 is targeted. It remains a controversial issue, with subsequent reports of attenuation of cocaine-enhanced extracellular NAc DA activity by CB2 agonists JWH133 and GW405833 in mice (258), but not by the pharmacological FAAH inhibitor URB597 in rats (259) adding to the complexity of the matter (see Table 2).

**Table 2:** **Pharmacological inhibition of FAAH inhibition and properties of psychostimulants**.

FAAH inhibitors	Drug	Animal	Outcome	Effects	Reference
AM404	Cocaine	Rat	Drug-induced lowering of brain reward/self-stimulation threshold	Impaired	Vlachou et al. (252)
			Drug-induced acute hyperlocomotion	Attenuated	Vlachou et al. (252)
URB597	Cocaine	Monkey	SA – effect of agonist after drug-taking extinction	No effect	Justinova et al. (260)
			SA – drug-taking behavior	No change	Justinova et al. (260)
		Rat	Cocaine-induced increase in VTA DA activity	Preserved	Luchicchi et al. (259)
			Cocaine-induced alterations in firing of NAc shell spiny neurons	Attenuated	Luchicchi et al. (259)
URB597, PMSF	Cocaine	Rat	SA – drug-seeking responses/intake	No change	Adamczyk et al. (261)
			SA – drug-induced reinstatement	Attenuated	Adamczyk et al. (261)
			SA – cue-induced reinstatement	Attenuated	Adamczyk et al. (261)

The increase of DA neurotransmission in the NAc and other striatal regions responsible for psychostimulant-induced rewarding parallels the stimulation of locomotor activity following acute drug administration. Sensitization to hyperlocomotor responses produced by psychostimulants occurs after chronic treatment, reflecting adaptive changes in DA transmission and potentially correlating with drug-seeking and reinstatement behavior (254, 262). In various studies, neither genetic deletion (262–264) nor pharmacological inhibition of CB1 receptors by SR141716A (265, 266) altered cocaine’s ability to induce acute motor effects or behavioral sensitization in rats and mice. However, a comparable number of reports described contradictory results, showing attenuation of both of these outcomes in CB1 knockout mice (253, 267) (see Table 3) as well as impairment of sensitization in animals pretreated with SR141716A (254, 268) or AM251 (267). Interestingly, although chronic cocaine use still induced sensitization in mice with invalidated CB1 receptors, sensitized response appeared somewhat changed when compared to control animals. Corbille et al. also found that AM251, unlike SR141716A (269), only impaired sensitization to cocaine after a single exposure, but not upon repeated administration. Similar experiments with cannabinoid agonists showed mixed results, as non-selective WIN 55,212-2 reduced cocaine’s motor effects, probably in a non-CB1 mediated fashion (252, 270). Likewise, CB2 agonists JWH133 and GW405833 decreased both acute hyperlocomotion and sensitization in rats (258), which parallels findings observed in mice genetically overexpressing CB2 (271). Δ^9^-THC failed to alter cocaine’s motor effects in rats (268, 272). Similarly, cannabinoid-amphetamine interactions studies demonstrate that acute cannabinoid exposure antagonizes amphetamine’s locomotion effects in a dose-dependent manner in rats. On the other hand, chronic exposure to Δ^9^-THC induces sensitization to the psychomotor effects mediated by amphetamine in rats (273). Taken together, these experiments suggest that the acute motor stimulant effects of psychostimulant and the induction of cocaine sensitization may not depend on endocannabinoid tone, even though CB1 receptors could play a minor modulating role in this regard [reviewed in Ref. (274)].

**Table 3:** **Effects of CB1 cannabinoid receptor deletion and properties of psychostimulants; CB1 receptor antagonists and properties of psychostimulants**.

Genotype	Drug	Animal	Outcome	Effects	Reference
CB1 KO	Cocaine	Mouse	Drug-induced acute hyperlocomotion	Preserved	Martin et al. (262), Houchi et al. (263), Miller et al. (264)
			Drug-induced acute hyperlocomotion	Attenuated	Corbille et al. (267), Li et al. (253)
			Drug-induced motor sensitization	Preserved	Martin et al. (262)
			Drug-induced motor sensitization	Attenuated	Corbille et al. (267)
			Drug-induced increase in NAc DA levels	Preserved	Soria et al. (255)
			Drug-induced increase in NAc DA levels	Attenuated	Li et al. (253)
			CPP – behavior acquisition under chronic unpredictable stress exposure	Preserved	Martin et al. (262), Houchi et al. (263), Miller et al. (264)
				Enhanced	Miller et al. (264)
			SA – behavior acquisition in restrained mobility protocol	Impaired	Soria et al. (255), Xi et al. (258)
				Preserved	Cossu et al. (275)
			SA – breaking point under PR schedule	Decreased	Soria et al. (255)
	Amphet.		Drug-induced acute hyperlocomotion	Preserved	Houchi et al. (263)
			Drug-induced acute hyperlocomotion	Attenuated	Corbille et al. (267)
			Drug-induced motor sensitization	Attenuated	Corbille et al. (267)
			SA – behavior acquisition in restrained mobility protocol	Preserved	Cossu et al. (275)

**Antagonist**	**Drug**	**Animal**	**Outcome**	**Effects**	**Reference**

AM251	Cocaine	Mouse	Drug-induced acute hyperlocomotion	Attenuated	Corbille et al. (267)
			Drug-induced motor sensitization (induction)	Attenuated	Corbille et al. (267)
			Drug-induced motor sensitization (expression)	Preserved	Corbille et al. (267)
			CPP – drug-induced reinstatement	Preserved	Vaughn et al. (276)
			CPP – stress-induced reinstatement	Impaired	Vaughn et al. (276)
		Rat	Drug-induced lowering of brain reward/self-stimulation threshold	Attenuated	Xi et al. (250)
			Drug-induced increase in NAc DA levels	Preserved	Xi et al. (257)
			Drug-induced increase in NAc glutamate	Attenuated	Xi et al. (257)
			SA – drug-induced reinstatement	Attenuated	Xi et al. (257), Adamczyk et al. (277)
			SA – cue-induced reinstatement	Attenuated	Adamczyk et al. (277)
			SA – drug-seeking responses/intake	No change	Xi et al. (250), Adamczyk et al. (277)
			SA – breaking point under PR schedule	Decreased	Xi et al. (250)
	METH	Rat	SA – drug-seeking responses/intake	Decreased	Vinklerova et al. (278)
SR141716A	Amphet., cocaine	Gerbils	Drug-induced acute hyperlocomotion	Decreased	Poncelet et al. (279)
			Reinstatement of drug-seeking	Decreased	Poncelet et al. (279)
	Cocaine	Monkey	SA – drug-seeking responses/intake	No change	Tanda et al. (280)
		Mouse	Drug-induced acute hyperlocomotion	Attenuated	Gerdeman et al. (268)
				Preserved	Lesscher et al. (266)
			Drug-induced motor sensitization (induction)	Attenuated	Gerdeman et al. (268)
				Preserved	Lesscher et al. (266)
			Drug-induced motor sensitization (expression)	Preserved	Gerdeman et al. (268)
			Drug-induced motor sensitization (maintenance – specific to a drug-paired environment)	Reversed	Gerdeman et al. (268)
			CPP – behavior acquisition	Impaired	Yu et al. (281)
			CPP – drug-induced reinstatement	Impaired	Yu et al. (281)
			SA – behavior acquisition	Preserved	Lesscher et al. (266)
			SA – “extinction burst responding”	Attenuated	Ward et al. (282)
			SA – time for behavior extinction	Decreased	Ward et al. (282)
			SA – cue-induced reinstatement	Attenuated	Ward et al. (282)
			SA – drug-seeking responses/intake	No change	De Vries et al. (283), Lesscher et al. (266)
			SA – breaking point under PR schedule	Decreased	Soria et al. (255)
		Rat	Drug-induced acute hyperlocomotion	Preserved	Chaperon et al. (265)
				Attenuated	Cheer et al. (254)
			Drug-induced motor sensitization (expression)	Attenuated	Filip et al. (269)
			Drug-induced lowering of brain reward/self-stimulation threshold	Preserved	Vlachou et al. (251), Xi et al. (250)
			Drug-induced decrease in VP GABA efflux	Preserved	Caille and Parsons (256)
			Drug-induced increase in NAc DA levels	Preserved	Caille and Parsons (256)
				Suppressed	Cheer et al. (254)
			Drug discrimination	Preserved	Filip et al. (269)
			SA – drug-seeking responses/intake	No change	De Vries et al. (283), Caille and Parsons (256), Filip et al. (269), Xi et al. (250)
			SA – breaking point under PR schedule	No change	Xi et al. (250)
				Decreased	Orio et al. (284)
			SA – drug-induced reinstatement	Attenuated	De Vries et al. (283), Filip et al. (269)
			SA – HU210-induced reinstatement	Attenuated	De Vries et al. (283)
			SA – cue-induced reinstatement	Attenuated	De Vries et al. (283), Filip et al. (269)
			SA – stress-induced reinstatement	Preserved	De Vries et al. (283)
			CPP – behavior acquisition	Impaired	Chaperon et al. (265)
			CPP – behavior expression	Preserved	Chaperon et al. (265)
	MDMA	Mouse	CPP – drug-induced reinstatement	Increased	Daza-Losada et al. (285)
			CPP – behavior acquisition	Impaired	Rodriguez-Arias et al. (286)
		Rat	CPP – behavior acquisition	Impaired	Braida et al. (287)
			SA – drug-seeking responses/intake	Increased	Braida and Sala (288)
	METH	Mouse	CPP – behavior acquisition	Impaired	Yu et al. (281)
			CPP – drug-induced reinstatement	Impaired	Yu et al. (281)
		Rat	Drug-induced reinstatement of drug-seeking behavior	Attenuated	Anggadiredja et al. (289)
			Cue-induced reinstatement of drug-seeking behavior	Attenuated	Anggadiredja et al. (289)

Overall, while some evidence of ECBS involvement in the neurobiological and behavioral correlates of psychostimulant reinforcing properties exists, influence of ECBS on acute psychostimulant reward is modest and probably involves a combination of mechanisms which may not directly involve DA activity in the NAc or CB1 receptors.

#### Influence of ECBS on acquisition and maintenance of psychostimulant-induced seeking behaviors

Models of conditioning such as the SA paradigm and the conditioned place-preference (CPP) procedure illustrate the reinforcing properties of drugs of abuse and demonstrate their ability to induce and maintain drug-seeking behaviors. Consistent with findings showing the limited involvement of the ECBS in the reinforcing properties of psychostimulants (see Influence of ECBS on Psychostimulants-Induced Behavioral and Reinforcing Effects), modulation of the ECBS appears to have a modest influence on acquisition and maintenance of psychostimulant-taking behavior in animals. In CPP experiments, while CB1 receptor deletion did not affect psychostimulant-induced place conditioning in mice, SR141716A impaired cocaine-, methamphetamine-, and MDMA-induced place conditioning in both rats and mice (262–265, 281, 286, 287). Difference in species, compensatory changes in the knockout animals due to the lack of CB1 receptors, as well as use of more intense conditioning and higher doses of drugs in the genetic deletion studies may have contributed to this discrepancy. This suggests that intensity of conditioning could overcome the effects of blocking ECBS signaling [reviewed in Ref. (274)]. It is important to note that the weaker cannabinoid antagonist CBD did not affect establishment of amphetamine-induced CPP in rats (290) and that the genetic overexpression of cannabinoid receptor CB2 impaired acquisition of both cocaine-induced CPP and SA (271). Thus the CPP model indicates that, although not directly interfering with the rewarding properties of psychostimulant drugs, ECBS could play a role in the perception and memory of psychostimulant reward, depending on environment-related factors.

In general, CB1 receptor invalidation does not seem to affect SA of psychostimulants. Experiments with genetic deletion of CB1 show conflicting results, as both knockout and SR141716A-treated mice still acquired amphetamine- and cocaine-taking behavior in restrained mobility conditions (266, 275), whereas knockout mice showed impaired SA behavior in other protocols (255, 258). Overall, results suggest that learning SA behavior might not require extensive ECBS involvement. Maintenance of such behavior may not depend on CB1 either, as drug-taking responses under a fixed-ratio schedule in animals that had already acquired cocaine SA remained unaffected after CB1 blockade by SR141716A in mice (266, 283), monkeys (280), and rats (250, 256, 269, 283) and by AM251 in rats (250, 277). Only one contradicting report exists in which AM251 decreased methamphetamine SA in conditioned rats (278). Cannabinoid signaling enhancement by the pharmacological FAAH inhibitors – URB597 and PMSF – also failed to affect maintenance of fixed-ratio drug-taking behavior in rats (261) and monkeys (260). On the other hand, cannabinoid stimulation by CB1 agonists had significant effects in several studies. Indeed, WIN 55,212-2 increased acquisition of MDMA SA in mice (286) and exposure to CP55,940 enhances development of cocaine SA in female rats (291). THC failed to alter acquisition of cocaine SA and amphetamine SA in monkeys (272) and rats (290), respectively. In regard to maintenance of drug intake in animals with SA behavior, cannabinoid agonists decreased drug-taking responses in rats – CP55,940 diminished MDMA intake (288) and WIN55,212-2 decreased cocaine administration (292) – and in monkeys – Δ^9^-THC also decreased cocaine intake (272). Fattore et al. first interpreted the shift in psychostimulant intake produced by CB1 agonists as indicative of a synergistic action of CB1 stimulation on reinforcing properties of the drugs, which, incidentally, could account for the frequent use of cannabis among human psychostimulants users (292). Complementary experiments using progressive-ratio schedules also reveal interaction of CB1 receptors with psychostimulant-induced reinforcing properties. In PR schedules, both genetic deletion and antagonist treatment of CB1 receptors produce a decrease in the maximal effort mice provided to self-administer cocaine, as made apparent by decreases in breaking point measures induced by SR141716A (255, 277, 284) and by AM251 (250) (SR141716A producing no effect in this specific report). This adds to the evidence that the CB1 receptors, while not indispensable for acquisition or maintenance of cocaine-seeking behavior, may exert a specific modulation on motivation and reward salience in psychostimulant addiction.

#### Role of ECBS in extinction and reinstatement of drug-taking behaviors

Although the mechanism used by endocannabinoid signaling to modulate psychostimulant reward and acquisition or maintenance is still the subject of debate, some form of consensus exists in the literature about the pivotal role of the ECBS in extinction and reinstatement in animal behavioral models of psychostimulant addiction [reviewed in Refs. (89) and (274)]. In conditioning procedures, extinction refers to the learning phase that follows the removal of the reinforcer (i.e., psychostimulant drugs), during which rates of conditioned responses (i.e., SA or CPP) progressively decline back to pre-conditioning levels. After drug-seeking behavior becomes extinct, several behavioral phenomena can reinstate drug-seeking behavior. These include not only re-exposure to the abused drug itself, but also exposure to contextual cues associated with previous drug administration and to environmental stressors (274, 293).

In a recent study from Ward et al. mice treated with SR141716 after removal of cocaine in a SA paradigm altered the burst in cocaine-seeking observed in the initial phase of extinction learning, while decreasing the time required to achieve complete extinction of cocaine-seeking behavior when compared to vehicle-treated mice (282). CB1 blockade by SR141716A also significantly decreases cue-induced reinstatement of cocaine SA behavior following extinction, supporting similar reports of attenuation of cue-induced reinstatement of cocaine-seeking by CB1 antagonism in rats (269, 277, 283). Evaluation of reinstatement of SA behavior induced by drug-priming produced similar results: SR141716A blocks cocaine-induced reinstatement (269, 283), and both AM251 (257, 277) and SR141716A inhibit methamphetamine-induced reinstatement (289). It is worth noting that CB2 antagonism also has an anti-reinstatement effect in cocaine-primed, but not in cue-exposed rats (277). In CPP models, CB1 blockade by SR141716A, but not by AM251 (276), impaired drug-induced reinstatement in cocaine-conditioned mice (281). SR141716A also impaired methamphetamine-induced reinstatement of CPP (281). Few studies focused on stress-induced reinstatement of psychostimulant-seeking. Vaughn et al. recently found that AM251 reverses stress-induced CPP (276). De Vries et al. could not find an impact of SR141716A on stress-induced SA reinstatement (283).

Stimulation of CB1 receptors produced opposite results to those obtained in pharmacological blockade experiments (see Table 4). WIN55,212-2 increases time for extinction of CPP and enhances drug-induced reinstatement in MDMA-conditioned mice (285, 286). Similarly, Δ^9^-THC increases cue-induced reinstatement of methamphetamine SA in rats (289). However, studies from Parker et al. and Adamczyk et al. complexify the interpretation of these results (290). Indeed, Adamczyk’s group showed that FAAH inhibition impairs cue- and drug-induced reinstatement of cocaine SA. Using the place-preference conditioning paradigm, Parker et al. have assessed the potential of both exogenous cannabinoids – Δ^9^-THC and CBD – to potentiate the extinction of cocaine- and amphetamine-induced CPP (290). After the establishment of cocaine-induced and amphetamine-induced place preference, rats were given an extinction trial, 30 min prior to which they were injected with a low dose of Δ^9^-THC, CBD, or vehicle. During conditioning trials, researchers also injected rats with cannabinoids, or vehicle, prior to an amphetamine injection, to determine the effects of Δ^9^-THC or CBD on the establishment and expression of a place preference. Results indicate that Δ^9^-THC and CBD potentiate the extinction of stimulant-CPP learning, which is not mediated by an alteration of learning or retrieval. CBD does not have a reinforcing or hedonic property on its own, suggesting that it does not have the addictive potential of Δ^9^-THC, a significant advantage in terms of therapeutic use. The non-reinforcing aspect of CBD has been replicated in studies looking at the co-administration of CBD and Δ^9^-THC (212, 294). These discrepancies probably result from the lack of receptor selectivity in the methods used to enhanced cannabinoid signaling. Nonetheless, these experiments support a significant involvement of ECBS in the extinction and reinstatement of behaviors related to psychostimulant addiction. Overall, the positive results of CB1 antagonists on extinction and prevention of reinstatement of psychostimulant SA, combined with their lack of reinforcing properties, suggest a therapeutic potential for CB1 modulation in treatment of psychostimulant addiction.

**Table 4:** **CB1 receptor agonists and properties of psychostimulants**.

Agonists	Drugs	Models	Outcome	Effects	Reference
CP55,940	Cocaine	Mouse	SA – effect of agonist after drug-taking extinction	No effect	Vaughn et al. (276)
		Rat	SA – behavior acquisition following exposure during adolescence in female specimen	Enhanced	Higuera-Matas et al. (291)
	MDMA	Rat	SA – drug-seeking responses/intake	Decreased	Braida and Sala (288)
HU210	Cocaine	Rat	SA – effect of agonist after drug-taking extinction	Reinstatement	De Vries et al. (283)
Δ^9^-THC	Amphet.	Rat	CPP – behavior acquisition	Preserved	Parker et al. (290)
			CPP – behavior extinction	Potentiated	Parker et al. (290)
	Cocaine	Monkey	SA – effect of agonist after drug-taking extinction	Reinstatement	Justinova et al. (260)
		Mouse	Drug-induced motor sensitization	Preserved	Gerdeman et al. (268)
		Rat	Drug-induced acute hyperlocomotion	Preserved	Panlilio et al. (272)
			Drug-induced motor sensitization	Preserved	Panlilio et al. (272)
			Drug-induced anxiety	Increased	Panlilio et al. (272)
			SA – behavior acquisition	Preserved	Panlilio et al. (272)
			SA – drug-seeking responses under PR schedule	Decreased	Panlilio et al. (272)
			CPP – behavior extinction	Potentiated	Parker et al. (290)
	METH	Rat	SA – cue-induced reinstatement	Increased	Anggadiredja et al. (289)
			SA – drug-induced reinstatement	Attenuated	Anggadiredja et al. (289)
WIN-55	Cocaine	Rat	Drug-induced acute hyperlocomotion	Attenuated	Przegalinski et al. (270), Vlachou et al. (252)
			Drug-induced lowering of brain reward/self-stimulation threshold	Impaired	Vlachou et al. (251)
			SA – drug-seeking responses/intake	Decreased	De Vries et al. (283)
	MDMA	Mouse	CPP – behavior acquisition	Increased	Rodriguez-Arias et al. (286)
			CPP – time for behavior extinction	Increased	Rodriguez-Arias et al. (286)
			CPP – drug-induced reinstatement	Increased	Rodriguez-Arias et al. (286), Daza-Losada et al. (285)

## Conclusion

A growing number of studies have investigated the neurobiological and behavioral mechanisms leading to psychostimulants dependence. A key feature of drug dependence is the relapse to drug use even after long period of abstinence. While greatly improved in recent years, treatment strategies for psychostimulants have yet to address effectively drug-seeking behaviors linked to high rates of relapse, persistent drug use as well as subsequent health, mental, and social problems. There is consequently an urgent need for researchers to identify compounds that might help patients (1) initiate abstinence and (2) avoid relapse. The ECBS appears to play a critical role in dependence to psychostimulants and experimental studies are now providing evidence that while it does not participate in the primary reinforcing properties of psychostimulants, it reliably modulates relapse to drugs. Interestingly, emerging human data supports a role for ECBS modulation in vulnerability to psychostimulant addiction, and more significantly in addictive behaviors among dependent individuals. Accumulating evidence thus points to the ECBS as a critical target for the development of pharmacotherapies for the treatment of addiction to psychostimulants. Given the various neuropharmacological actions of exogenous cannabinoids, and their ability to modulate the acute reinforcing effects of drugs, data on Δ^9^-THC and CBD is particularly promising as to the potential use of cannabinoids in relapse prevention strategies for psychostimulant-dependent individuals. The effects of these compounds on stimulant use outcomes in humans remains to be clearly established and could be assessed with well-designed controlled trials. The neurobiological correlates of cannabinoids’ impact on stimulant-seeking behaviors could also be examined with neuroimaging studies in stimulants dependent individuals. Among potential barriers, social and scientific acceptability of cannabinoid-based therapy, side effects profiles, as well as addictive potential of certain cannabinoid such as Δ^9^-THC, have to be taken into consideration.

## Conflict of Interest Statement

The authors declare that the research was conducted in the absence of any commercial or financial relationships that could be construed as a potential conflict of interest.
